# The Mycobiome: Cancer Pathogenesis, Diagnosis, and Therapy

**DOI:** 10.3390/cancers14122875

**Published:** 2022-06-10

**Authors:** Ahmed Gamal, Mohammed Elshaer, Mayyadah Alabdely, Ahmed Kadry, Thomas S. McCormick, Mahmoud Ghannoum

**Affiliations:** 1 Department of Dermatology, Case Western Reserve University, Cleveland, OH 44106, USA; axg1117@case.edu (A.G.); melshaer85@mans.edu.eg (M.E.); ake27@case.edu (A.K.); tsm4@case.edu (T.S.M.); 2 Department of Clinical Pathology, Faculty of Medicine, Mansoura University, Mansoura 35516, Egypt; 3 Department of Internal Medicine, University Hospitals Cleveland Medical Center, Cleveland, OH 44106, USA; mayyadah.alabdely@uhhospitals.org; 4 Department of Dermatology and Venereology, Al-Azhar University, Cairo 11651, Egypt; 5 Department of Dermatology, University Hospitals Cleveland Medical Center, Cleveland, OH 44106, USA

**Keywords:** mycobiome, fungi, cancer, cancer pathogenesis, anticancer, anticancer, cancer therapy

## Abstract

**Simple Summary:**

The role of the microbiome in health and disease has gained great attention over the past few decades. The evidence demonstrating the link between the microbiome and cancer has been growing. Although several studies have explored this topic, their main focus has been on bacteria, ignoring another important group in the microbiome: fungi. Recent studies have demonstrated the complex interactions between bacteria and fungi inhabiting the human body. Furthermore, published data show that the mycobiome has a significant role in the development of several cancers, such as the link between *Malassezia* and pancreatic duct adenocarcinoma. Additionally, studying bacteria and fungi together was shown to be more informative compared to single community analysis. Similarly, bacteriome and mycobiome modulation have significant positive effects on the efficacy and tolerability of the available anticancer therapies. Thus, microbiome studies should also include other microbial communities, such as fungi.

**Abstract:**

Cancer is among the leading causes of death globally. Despite advances in cancer research, a full understanding of the exact cause has not been established. Recent data have shown that the microbiome has an important relationship with cancer on various levels, including cancer pathogenesis, diagnosis and prognosis, and treatment. Since most studies have focused only on the role of bacteria in this process, in this article we review the role of fungi—another important group of the microbiome, the totality of which is referred to as the “mycobiome”—in the development of cancer and how it can impact responses to anticancer medications. Furthermore, we provide recent evidence that shows how the different microbial communities interact and affect each other at gastrointestinal and non-gastrointestinal sites, including the skin, thereby emphasizing the importance of investigating the microbiome beyond bacteria.

## 1. Introduction

Cancer is among the leading causes of death globally, accounting for nearly 10 million deaths in 2020 [1]. Multiple factors play a role in cancer development, including genetic and environmental influences, chronic inflammation, and infections (e.g., human papillomavirus). Regarding the role of infection with pathogenic organisms, it has been suggested that an imbalance (or dysbiosis) in the abundance of commensal organisms inhabiting various sites of the body (the microbiome) may lead to cancer development and progression [2]. Although this is a nascent area of research, several direct and indirect mechanisms have been described in the literature demonstrating increases or decreases in certain microbes associated with pro-inflammatory responses, epithelial cell transformation, and DNA damage, all of which have a pro-carcinogenic effect [3].

In the past few decades, most microbiome studies have focused on identifying bacteria in the gut that were associated with dysbiosis and had potential links to chronic disorders. However, recent studies have begun to address the need to expand the scope of microbiome studies to include other microbial communities, including fungi, viruses, and protozoa [4,5]—in other words, expanding microbiome studies beyond bacteria. Moreover, recent studies showed that these microbial communities actively interact, and these polymicrobial interactions have been demonstrated to affect the health of the host and modify inter-kingdom interactions [6,7].

Fungal communities that reside in and on our bodies are collectively referred to as the mycobiome. The mycobiome is often neglected as a potential cause of disease, as it is comparatively less abundant (<0.1% of total microbiota) and less diverse, although fungi are much larger than bacteria and exhibit metabolic gene clusters that correlate with different ecological needs [8]. In comparison to the bacteriome, there are limited data regarding the mycobiome. Fortunately, the recent utilization of novel genomic sequencing in fungal research has expanded our knowledge regarding their contributions to health and disease [9].

The mycobiome can be found at different anatomical sites, including the skin [10], lungs [11], oral cavity [12], vagina [13], and gastrointestinal tract [14]. There are distinctive fungal communities occupying the various anatomical sites [15]. Most studies have suggested that the *Ascomycota* phylum, which includes *Candida* spp., *Cladosporium* spp., and *Saccharomyces cerevisiae*, is the most dominant phylum, accounting for >90% relative abundance at various body sites, followed by *Basidiomycota* and Zygomycota phyla [10,12,16,17].

In healthy individuals, fungi play a major role in normal host immune response and homeostasis [18]. They are recognized by multiple receptors located on antigen-presenting and natural killer cells, resulting in the formation of proinflammatory mediators, such as interleukins, tumor necrosis factor-α (TNF-α), and interferon-gamma (IFN-γ) [19]. Furthermore, fungal probiotics exhibit anti-neoplastic properties, similar to the protective effects of bacterial probiotics and prebiotics, and may be useful in cancer prevention and treatment. In addition, *S. cerevisiae*, *S. boullardii* (*S. cerevisiae* variant), and *Schizophyllum commune* have been shown to have antioxidant and other beneficial effects, including inhibition of pathogenic bacteria and yeast [20].

In this article, we review the role of the human mycobiome in carcinogenesis, highlighting the interplay between the human mycobiome and bacteriome, possible future diagnostic tools, and the potential for the development of novel therapeutics (Table 1).

## 2. Role of the Mycobiome in Cancer Pathogenesis

### 2.1. Microbial Inflammation

**Figure 1:**
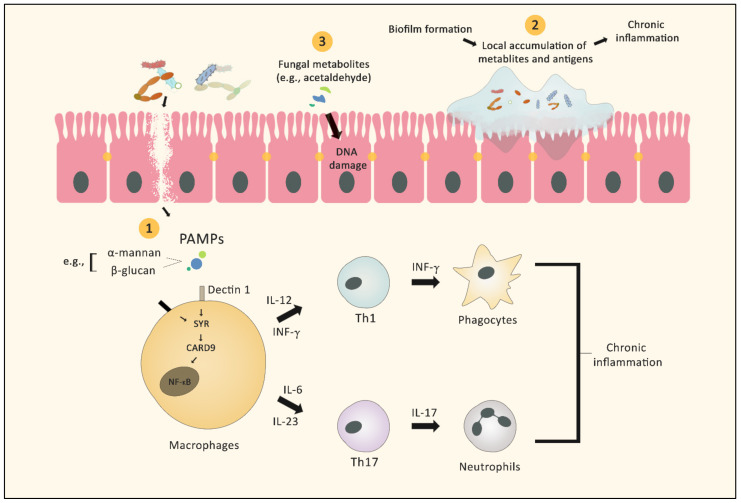
Graphic illustration of the different mechanisms by which fungi (with or without bacteria) can facilitate the development of cancer. 1. Fungal antigens act as PAMPs stimulating several signaling cascades, leading to activation of neutrophils and macrophages, which in turn results in chronic inflammation. 2. Mixed species bacterial–fungal biofilms act as a barrier that protects the microbes from the immune system and maintains the local inflammatory reaction. 3. Fungal metabolites can exert direct carcinogenic effects, such as DNA damage.

Inflammation is widely believed to contribute to cancer development and progression, as indicated by higher cancer incidence among individuals with various chronic inflammatory disorders, such as IBD, ulcerative colitis, pancreatitis, and chronic atrophic gastritis [34]. Several studies have focused on the heightened immune response following fungal infection [35]. During fungal invasion, pattern recognition receptors (PRRs) identify pathogen-associated molecular patterns (PAMPs) comprised of the carbohydrate components of the fungal cell wall. Pattern recognition receptor binding of PAMPs initiates signaling cascades through several pathways. These pathways include Toll/IL-1R domain-containing adaptor-inducing IFN-β (TRIF), spleen tyrosine kinase—caspase recruitment domain 9 (SYK-CARD9), and myeloid differentiation primary response gene 88 (MYD88). Initiation of these pathways results in the synthesis of various signaling molecules, including interleukin 1β (IL-1β), IL-6, IL-12, IL-23, transforming growth factor-β (TGF-β), and interferon -γ (IFN-γ), which orchestrate the antifungal response mediated by Th1 and Th17 cells, in coordination with phagocyte activation and neutrophil recruitment [36]. (Figure 1).

Many PRRs participate in the control of the innate immune response and the maintenance of the integrity of epithelial barriers. Breakdown of barriers due to the inadequate activity of PRR signaling is a primary cause of microbial-derived tumorigenesis [37]. For example, CARD9 is a signaling protein that activates the NF-ĸΒ and mitogen-activated protein kinase (MAPK) pathways, resulting in the release of pro-inflammatory cytokine cascades [38]. An inherited deficiency of CARD9 leads to reduced fungicidal activity of macrophages, resulting in a higher fungal load, especially with *Candida tropicalis*. Interestingly, *C. tropicalis* was reported to trigger the recruitment of myeloid-derived suppressor cells (MDSCs), which induce immunosuppression and tumorigenesis by increasing the local production of nitric oxide, reactive oxygen species, and cytokines. Wang et al. found that CARD9 expression in the colon was affected by the colonic fungal burden. In colorectal cancer (CRC) patients, a low fungal burden was related to relatively higher CARD9 expression, and vice versa. Additionally, *C. tropicalis* had higher abundance in CRC patients compared to healthy individuals [39].

MYD88 is an important PRR that regulates the integrity of the intestinal epithelial barrier. Knockout of *Myd88* in mice (Myd88^−/−^*)* caused a lack of mucosal repair ability following injury. This in turn altered the inflammatory environment, exacerbating the mutation rate of mucosal epithelial cells, and consequently increasing adenoma formation and cancer progression [40]. MYD88 is also an IL-18 signaling mediator, whose absence may promote cancer by inhibiting the activity of an IL-18-dependent pathway. Ulcerative colitis (UC) is a well-known clinical example of barrier dysfunction, in which an intestinal barrier defect contributes to disease progression and increases the risk of cancer [41].

Another example of spontaneous development of CRC caused by a malfunctioning epithelial barrier was observed in *MUC2* deficient mice. The *MUC2* gene product is linked to the protective mechanism of intestinal epithelial surfaces against the contents of the gut lumen, including the microbial communities. Therefore, loss of this protection generates a mild, chronic inflammatory environment that may lead to increased intestinal microbiota—mucosa contact and eventually the development of carcinomas, as shown in preclinical murine models [42].

Additional examples of the downstream effects of barrier disruption are demonstrated by the release of a cytolytic toxin termed candidalysin from *C. albicans.* Increased candidalysin was shown to facilitate the translocation of *C. albicans* across intestinal epithelia [43]. This toxin has the potential to adversely affect epithelial barrier function and modulate the immunological response via the release of proinflammatory mediators such as IL-1, IL-6, IL-8, and TNF-α, and induction of a Th17 response that acts indirectly by neutrophil recruitment towards the tumor site and correlates with poor disease prognosis [36]. Additionally, the underlying mechanism by which candidalysin caused immune stimulation included activation of the epidermal growth factor receptor (EGFR) indirectly through its effect on matrix metalloproteinases (MMPs) and EGFR ligands, both of which have been independently linked to several cancers [44,45,46]. *C. albicans* also has the ability to activate epithelial MAPK and extracellular signal-regulated kinase (ERK) signaling pathways, which both play a role in tissue growth, loss of E-cadherin and occludin, and activation of angiogenesis [47,48,49,50,51].

Interestingly, translocation of fungal species from the intestinal lumen to the pancreas, particularly *Malassezia*, has been reported to induce pancreatic ductal adenocarcinoma. The underlying mechanism for this includes engagement of mannose-binding lectin (MBL) with the fungal cell wall glycans, resulting in activation of the complement cascade. Complement activation is hypothesized to participate in cancer development and be necessary for oncogenic progression; and MBL, or complement 3 (C3) deletion in the extra-tumoral compartment, or C3aR knockdown in tumor cells, was protective in murine studies [26].

### 2.2. Biofilm Formation

Studies have emphasized the importance of bacterial biofilms in a wide variety of medical conditions [52,53,54]. However, recent studies have shown that bacterial and fungal biofilms are equally important. For instance, we previously demonstrated that fungi and bacteria interact to produce biofilms capable of exacerbating intestinal inflammation [55]. Importantly, some of the most compelling evidence demonstrates that colonic biofilms can cause colorectal cancer in mice [56]. As reported by Dejea et al., inoculation of different types of murine models with homogenates of colonic biofilms resulted in promotion of colorectal cancer compared to controls (i.e., without biofilms). Moreover, scanning and transmission electron (SEM, TEM) microscopy showed that within a polymicrobial biofilm, *E. coli* cells can fuse to the candidal cell walls, and *Serratia marcescens* can form a “bridge” between *C. tropicalis* and *E. coli* via fimbriae. This close bacterial–fungal interaction serves as a powerful barrier protecting the microorganisms against killing by the host, in addition to providing resistance to antimicrobials, resulting in a persistent inflammatory reaction [57,58]. Similarly, *C. albicans* develops biofilms that protect the fungi from environmental factors and are associated with inadequate clearance by the host immune system. Moreover, *C. albicans* exacerbates dextran sodium sulfate (DSS)-induced colitis in mice when co-colonized with *Enterobacteriaceae* such as *E. coli* [57]. These observations suggest that fungi, in the presence of a particular bacterial environment, may exert similar effects in CRC patients.

*Fusobacterium nucleatum*, an opportunistic bacterial pathogen that resides in the human mouth and gastrointestinal tract, was shown to contribute to the development of CRC through various mechanisms, such as binding to immune cells causing immunosuppression, and recruiting tumor-infiltrating immune cells, causing a pro-inflammatory microenvironment, which promotes CRC progression [59]. *F. nucleatum* was also shown to co-aggregate with *C. albicans* via the interaction of genetic and structural cellular components [60]. This aggregation synergistically benefits colonization of the oral cavity and gastrointestinal tract, thereby enhancing pathogenesis and maintaining local inflammation, which facilitates tumorigenesis [20].

Fungal–bacterial interactions have been widely reported in the literature, often with clinical significance. Recently, these interactions have gained attention due to their impacts on human health [61]. Understanding the nature of these interactions is the key for the prevention and management of polymicrobial infections and channeling them to gain potential beneficial effects. An imbalance between these microbes (also known as “dysbiosis”) may predispose the host to a variety of chronic fungal infections and diseases at local and distant sites [62,63]. Moreover, having a better understanding of such interactions may be helpful in the identification of novel targets for future anti-cancer treatments [64].

Several lines of evidence reported in the literature address these types of interactions, such as quorum-sensing molecules from the bacterium *Pseudomonas aeruginosa* that can alter the morphology of *C. albicans* in both liquid and solid media [65]. Another example is the synergy between *E. coli* and *C. albicans* that resulted in the death of mice significantly faster than those infected with *C. albicans* alone [66].

Bacteria and fungi may interact synergistically or antagonistically. Examples of synergy between gut bacteria and fungi that may predispose patients toward cellular oncogenesis include: enterohemorrhagic *E. coli* promote the invasiveness and tissue damage of enterocytes infected with *C. albicans* in vitro [67]; [68] *C. albicans*-mediated enhancement of strictly anaerobic *Clostridium difficile* due to a reduced oxygen level within the yeast’s vicinity [69]; and *Helicobacter pylori* survival in *C. albicans* vacuoles at reduced pH [70]. Examples of antagonistic effects between fungi and bacteria include: (1) the antifungal activity of some bacterial species such as *Acinetobacter baumanii*, *Serratia marcescens*, and *Salmonella typhimurium* against *Candida* spp. [71]; and [68] p-cresol’s (produced by *Clostridium difficile*) inhibitory effect on the growth of *C. albicans* [72]. It is important to consider such interactions when developing probiotic-based therapeutics for prevention or treatment of cancer [71].

### 2.3. Fungus-Derived Metabolites and Cancer

Several studies have demonstrated the ability of various *Candida* species to produce acetaldehyde by their alcohol dehydrogenase enzyme [73,74,75]. Acetaldehyde is a substance that has been shown to promote carcinogenesis [76]. The carcinogenic effect of acetaldehyde was clearly demonstrated in aldehyde dehydrogenase-2 (an enzyme that helps the human body to eliminate acetaldehyde by oxidizing it into acetate) deficient Asian subjects that were shown to have increased levels of acetaldehyde in their saliva, and a 10-fold increased risk of oral cancer compared to individuals with normal enzyme function [77]. Although mitochondrial aldehyde dehydrogenase-2 can help in the elimination of acetaldehyde, dysbiosis with overgrowth of *Candida* may result in a significant increase in its production, especially in heavy alcohol consumers, thus increasing the risk of oral cancer development [78,79]. Furthermore, *C. albicans* was suggested to participate in metabolizing glucose to carcinogenic acetaldehyde in the mouth, which may also promote oral cancer in non-alcohol drinkers [74]. It is important to mention that the role of alcohol dehydrogenase in cancer development is not limited to the production of acetaldehyde. Reduction of alcohol dehydrogenase enhances *Candida* biofilm formation, another potential mechanism for cancer promotion, as discussed in the section above [80]. Interestingly, some studies have demonstrated the ability of *Candida* to invade tissue and produce nitrosamines, compounds that may activate specific proto-oncogenes responsible for malignant transformation [81].

Toxin production by intestinal microbiota has been suggested to increase the risk of cancer through various mechanisms. For example, *Aspergillus* can contribute to hepatocellular cancer by excreting metabolic products known as aflatoxins, of which aflatoxin B1 (AFB1) is the most hepatocarcinogenic mycotoxin. AFB1 can cause cancer by the formation of DNA adducts, DNA strand breakage, and oxidative damage in target hepatocytes [82,83,84]. Another *Aspergillus* product linked to cancer is patulin toxin, which is found in some fruits, such as apples, and their derived by-products [85]. Besides its systemic effects, patulin has the potential for causing DNA damage by stimulating the formation of reactive oxygen species which may facilitate carcinogenesis [86,87]. This was demonstrated by Saxena et al., who showed that a single topical application on the skin of mice followed by a twice-weekly application of 12-tetradecanoyl phorbol myristate acetate [88] resulted in the development of squamous cell carcinoma after 14 days [89]. Notably, no tumors were observed when patulin was used alone on the skin in the same study, suggesting that a two-hit stimuli scenario is necessary. Despite these observations, studies on the effect of this toxin on humans are very limited, and its carcinogenic potential is still unproven.

Another microbiome-associated toxin linked to oncogenesis is fumonisin B1, a toxin produced by *Fusarium* mold species and connected to esophageal cancer by intake of corn grains containing the toxin [90]. Potential mechanisms by which this toxin can facilitate the development of cancer include being able to decrease cell viability and proliferation in a concentration-dependent manner by causing accumulation of sphinganine, which halts cells at the G0/G1 phase, resulting in growth inhibition and apoptosis [91,92].

## 3. Diagnostic Implications of the Mycobiome in Cancer Patients

Recent studies have demonstrated distinct value for mycobiome analysis in predicting the likelihood of an individual having a certain disorder, including cancer and non-cancer-related conditions, such as irritable bowel syndrome [8,21,22,93]. However, limited studies have investigated the use of fungal profiling as a diagnostic or predictive tool for cancer. Evaluating whether members of the mycobiome have utility in predicting cancer is warranted. A summary of this section is found in Table 2.

### 3.1. Cancers of the Gastrointestinal Tract

Analysis of the microbiome of CRC patients, including the fungal community, has shown a pattern of alteration common among studies. For example, fecal mycobiota analysis, from a total of 131 subjects, including 74 CRC patients, 29 colon-polyp patients, and 28 healthy controls, showed that there was a significant increase in the *Ascomycota*: *Basidiomycota* ratio in CRC and polyp patients compared with the controls [22]. Moreover, enrichment of *Microascaceae* and *Sordariaceae* spp. was observed in CRC and polyp cohorts compared to healthy controls. In addition, the stage of disease had an impact on the mycobiota population, as noted by the higher fungal diversity observed in late-stage CRC patients compared to early-stage patients. In this regard, significant increases in *Microbotryomycetes*, *Sordariomycetes*, *Microascaceae*, *Sordariales*, *Lasiosphaeriaceae*, and *Microascales* were detected; and decreases in the abundance of *Pleosporaceae* and *Alternaria* were noted [22].

Similarly, Luan et al., who analyzed intestinal biopsy samples from adenomas and adjacent tissue from 27 patients, reported that besides a significant decrease in the diversity of fungal communities in adenomas compared to adjacent tissue, *Basidiomycota* was significantly enriched in adjacent biopsy samples, and the phylum Chytridiomycota was significantly enriched in adenomas compared with adjacent biopsy samples. Furthermore, order *Saccharomycetales* and phylum *Basidiomycota* organisms were significantly enriched in advanced compared to non-advanced adenoma tissue biopsies [21].

In agreement with these findings, a study conducted in China showed that the abundances of *Candida*, *Saccharomyces*, and *Ascomycete* were elevated in patients with colonic polyps compared to controls [94]. Furthermore, Coker et al. reported considerable enrichment of *Rhodotorula* and *Malassezia* of the *Basidiomycota* phylum and *Acremonium* of the *Ascomycota* phylum in fecal samples from CRC patients compared to controls, whereas *Saccharomycetes*, especially *S. cerevisiae*, were found to be depleted [8]. Interestingly, Chin et al. reported distinctive sets of proteins secreted by *Schizosaccharomyces pombe* in the stool samples of CRC patients, and four proteins (SWR1 complex bromodomain subunit bdf1, structural maintenance of chromosomes protein 5, DNA repair protein rhp57, and uncharacterized WD repeat-containing protein C16H5.13) were closely linked with advanced CRC [24].

In both animal models and human studies, pancreatic ductal adenocarcinoma (PDA) was found to have a 3000-fold increase in fungal abundance compared to a cohort of subjects with healthy pancreases [26]. Aykut and colleagues assessed the mycobiomes of patients with PDA using 18S rRNA sequencing and reported a distinct mycobiome composition compared to subjects with normal pancreases, suggesting fungal dysbiosis during tumorigenesis. The use of antifungal medication in a mouse model of PDA protected against tumor progression, whereas repopulation with *Malassezia*, the most common fungal genus in PDA, resulted in rapid growth of pancreatic tumors. Repopulation with other fungal species such as *Candida* and *Aspergillus* did not impact the tumor growth, suggesting that the effect is *Malassezia* specific [26].

Recently, there came evidence that an altered mycobiome profile within the oral cavity has a potential role in development of oral squamous cell carcinoma (OSCC). In a study by Makinen et al., *C. albicans* was the most commonly isolated species among OSCC in most patients. *C. dubliniensis*, *C. tropicalis*, *C. glabrata*, *and C. parapsilosis* were isolated in small portions of patients [31]. Moreover, Shay et al. demonstrated that *Malassezia* was reduced in abundance in patients with head and neck SCC (HNSCC) [30]. In a separate study, Banerjee et al. analyzed the mycobiomes of 100 samples of OSCC compared to those of 20 adjacent healthy control tissues (obtained from the cancer patients enrolled in the study) and 20 oral tissues from other non-cancer healthy individuals. *Rhodotorula*, *Geotrichum*, and *Pneumocystis* were detected only in samples obtained from the cancer patients. Additionally, *Fonsecaea* was found in both cancer samples and cancer-adjacent tissue from the same patients, but not in oral tissue from healthy subjects [95]. Moreover, as reported by Mukherjee et al., 39 paired tumor and adjacent normal tissues from patients with SCC of the tongue demonstrated distinct differences in bacteriome and mycobiome between cancer and healthy control cohorts [96]. There were significant reductions in the bacterial diversity and richness and fungal richness in tumor tissue compared to the matched non-tumor tissues (*p* < 0.006). Glomeromycota was the only fungal phylum that was significantly decreased in the tumor group compared to the matched non-tumor tissues. Interestingly, high T-stage tumor samples exhibited increased richness of both bacteria and fungi at the phylum level compared to low T-stage samples (*p* ≤ 0.047).

It is important to note that the results of these studies have been variable, showing no causal relationship between tumor development and mycobiome profiles. Thus, this area of research requires further investigation with unified methodologies to address the potential limitations of these types of studies.

Several studies have examined potential connections between oral candidiasis and the development of head and neck cancer (HNC) [28,29,96]. In comparison to benign tissue, Perera et al. reported an overgrowth of *C. albicans* in oral squamous cell malignant tissue [28]. Vesty et al., also detected a higher concentration of *C. albicans* in the saliva of HNSCC patients, which was associated with production of increased inflammatory cytokines [29]. It was suggested that *C. albicans* may contribute to carcinogenesis by hyper-methylation of tumor suppressor genes. Conversely, the presence of *C. albicans* in both healthy individuals and cancer patients is insufficient proof that this organism is responsible for the development of HNSCC [100,101,102].

### 3.2. Cancers beyond the Gastrointestinal Tract

Associations between fungi and cancer in non-gastrointestinal organs have been previously reported. For example, Banerjee et al. analyzed the microbiome signatures of various tumors, including ovarian and breast cancer tumors [97,98]. Analysis of the microbiome associated with ovarian cancer showed significant alterations in microbial communities, including fungi [97]. Interestingly, 18 fungal signatures were detected only in ovarian cancer samples and not controls (i.e., non-cancerous tissue). These signatures included *Cladosporium*, with the highest hybridization signal, *Acremonium*, and *Candida*. Additionally, the prevalence of *Malassezia* was high in all ovarian cancer samples screened; however, this was also observed in matched controls [97]. In addition, microbiome analysis of various types of breast cancer samples, classified based on the receptors expressed on their surfaces, including estrogen receptor-positive (ER+), Her2 receptor-positive (HR+) triple positive (TP; estrogen, progesterone, and Her2 receptor-positive), and triple-negative (TN; estrogen, progesterone and Her2 receptor negative) [98] showed the highest fungal diversity in ER+ samples; and HR+ samples had the least fungal diversity. *Arthroderma* accounted for the highest average hybridization signal. Additionally, *Candida*, *Cryptococcus*, *Mucor*, *Penicillium*, *Trichophyton*, and *Trichosporon* were observed in all cancer samples [98].

The link between skin cancers and bacteria and viruses has been extensively discussed, although limited attention has been paid to another important part of the human microbiome, the fungi. In this section, we discuss a few of the studies that explored the possible role that fungi play in skin cancer.

Vitali et al. characterized the gut fungal community in addition to the bacterial community in skin cancer patients [99]. In 20 subjects diagnosed with melanoma, the α-diversity of the fungal community revealed significantly higher richness in melanoma patients compared to healthy individuals and showed that the order *Saccharomycetales* was enriched in melanoma patients. However, as the disease progressed, a reduction in alpha diversity was noted (dysbiosis). Principal coordinate analysis (PCA) of the gut microbiome showed no significant difference between melanoma patients and healthy individuals when each community was analyzed separately (i.e., fungal and bacterial). However, analysis of both communities combined revealed a significant difference between both groups. Another important finding of the Vitali et al. work was that the gut microbiota were significantly different between patients with different stages of melanoma (i.e., in situ versus invasive melanoma). One hundred and eighty (180) zero-radius operational taxonomic units (zOTUs) were enriched in melanoma patients (117 bacterial zOTUs and 63 fungal zOTUs). Out of these zOTUs, 162 were mainly enriched in patients with in situ melanoma. Interestingly, out of 23 zOTUs that were enriched in patients with and without melanoma in regression, 16 were specifically increased in the regression group. Furthermore, analysis of the gut microbiota revealed a significant difference between metastatic and non-metastatic melanoma patients. Combined, these data strongly support the potential use of mycobiome and bacteriome analysis as a diagnostic and/or prognostic tool. Finally, this approach emphasizes the importance of analyzing both communities together, which is reasonable given that different microbes inhabiting the human body interact and affect each other, as described above [71].

This concept is also supported by the work of Shiao and colleagues, who tested the effect of microbiome alteration on tumor radiotherapy efficacy [103]. The use of antibiotics to deplete the bacteriome in C57BL/6 mice prior to subcutaneous inoculation with 1 × 10^7^ B16 murine melanoma cells resulted in a massive increase in the fungal order *Saccharomycetales*, especially *C. albican* and *Saccharomyces* genera. The shift in the bacterial/fungal balance was associated with a reduction in the anticancer immunity. In the same study, heavy colonization of mice with *C. albicans* resulted in an increased proportion of PD-1^+^ CD8^+^ T cells, which are known to cause a more immunosuppressive tumor microenvironment, and increased tumor regrowth was observed [103].

Thus, although the direct link between the fungal elements of the human microbiota and melanoma is still unknown, there is clear evidence that fungi can modulate the tumor microenvironment via immune modulation that may enhance or suppress tumor growth and treatment response.

## 4. Mycobiome and Cancer Therapy

The microbiome’s influence over cancer is not restricted to cancer pathogenesis. Several studies have shown that the microbiome and mycobiome may positively or negatively affect the patient’s response to anticancer therapy (i.e., chemotherapy, immunotherapy, or radiotherapy). The microbiome can also be affected by anticancer therapies, in turn altering the course of disease [104,105,106]. Thus, there is a great opportunity to influence the course of cancer through modulation of the gut mycobiome and bacteriome, which can be achieved by several modalities, such as diet, probiotic/prebiotic, or fecal microbiota transplantation (FMT). In the following section, we highlight evidence of how the mycobiome can alter the responses to anticancer therapies, along with the impacts of anticancer therapies on the mycobiome and the significance of these disruptions (Table 3).

### 4.1. Effect of Mycobiome on Anticancer Therapy

Several studies have demonstrated that the gut microbiota can enhance or impair the efficacy of commonly used chemotherapeutic agents, such as irinotecan, oxaliplatin, cyclophosphamide, 5-fluorouracil, gemcitabine, and anthracyclines [122,127,128,129,130]. Moreover, emerging evidence suggests that gut-resident bacteria and fungi may influence the anticancer response during immunotherapy by priming innate and adaptive immune responses and increasing the cytokine production by antigen-presenting cells or lymphocytes [26,103,131,132,133,134,135,136]. Here we review the available literature that supports this hypothesis and the possible future therapies.

#### 4.1.1. Tumor Microenvironment Modification

As one of the most abundant polysaccharides in the fungal cell wall, β-glucan has been postulated to exhibit anticancer activity. Based on recent experimental studies, systemic administration of certain β-glucans effectively regulates the tumor microenvironment (TME), resulting in a considerable reduction in tumor growth and distant metastases [137]. These findings imply that β-glucans are potent immunomodulators capable of altering innate and adaptive immune responses in the TME and enhancing the overall response to existing cancer immunotherapies.

Additionally, oral administration of β-glucan can boost phagocytes’ tumoricidal activity against iC3b-opsonized cancer cells. The proposed underlying mechanism is that oral β-glucan is engulfed by intestinal macrophages and transported to the bone marrow, where it is digested and released as smaller fragments that granulocytes engulf via complement receptor 3 (CR3). The CR3-primed granulocytes can attack iC3b/mAb-coated tumor cells [107]. Orally administered β-glucan has also been postulated to decrease the tumor burden by inducing the transition of immunosuppressive M2 macrophages into inflammatory M1 macrophages [68,108].

In a prospective trial that included 23 female patients with advanced breast cancer, oral administration of two 10 mg capsules of soluble β-glucan derived from *S. cerevisiae* enhanced the proliferation and activation of peripheral blood monocytes and caused no clinical adverse effects [109]. Although the mean leukocyte count was not changed significantly, the mean monocyte count increased from 326 ± 124/mm^3^ to 496 ± 194/mm^3^ on day 15 post-administration (*p* = 0.015). Moreover, a slight increase in the absolute percentage of circulating monocytes was also observed (7.4% vs. 12%) after 14 days of β-glucan therapy (*p* = 0.003). This suggests that β-glucan may possess an immunomodulatory effect that can help improve responsiveness to available anticancer therapies. This hypothesis could be supported by the reported increase in CD95 expression on CD14+ monocytes observed by Demir et al. (48.17% at initiation vs. 69.23% 15-days after β-glucan supplementation (*p* = 0.002) [109]. CD95 is a class of cell death receptors that regulates immune responses by transducing apoptotic signals that induce programmed cell death and stimulating macrophages to produce high amounts of proinflammatory cytokines, such as TNF-α [138].

In mice inoculated with a highly metastatic cell line of colon 26 carcinoma (colon 26-M3.1) or B16-BL6 melanoma cells, intravenous treatment with β-glucan derived from mutated *S. cerevisiae* strain (i.e., zymolase resistant *S. cerevisiae*) suppressed cancer cell proliferation in a dose-dependent manner. Additionally, pretreatment of mice with the same type of β-glucan, 2 days before inoculation with the tumor cells, enhanced the mice’s survival time. These effects were also associated with enhanced pro-inflammatory cytokine production and anticancer activity of peritoneal macrophages, and increased natural killer (NK) cell cytotoxicity [110]. Interestingly, β-glucan derived from wild-type *S. cerevisiae* was five times less effective than β-glucan derived from mutated *S. cerevisiae* at inhibiting lung metastasis. Mutated *S. cerevisiae* β-glucan had the ability to inhibit tumor metastasis produced by both hematogenous and non-hematogenous tumor cells, although the effect was dependent on the tumor cell type. These observations were in agreement with data produced by Kim and colleagues: they found that prophylactic intravenous administration of β-glucan derived from mutated *S. cerevisiae* in the same animal model, in combination with cisplatin, had better efficacy compared to the chemotherapy agent alone [111]. Similarly, in an experimental study by Vetvicka et al., insoluble β-glucan extracted from *S. cerevisiae* suppressed the development of melanoma in a dose-dependent manner [112].

Systemic administration of oat-derived β-(1-3)–(1-4)-glucan of 200 kDa molecular mass (BG34-200) in mice by Zhang et al. demonstrated that the immunogenic milieu of the melanoma microenvironment was changed, allowing M1-type macrophage activation. This in turn caused the production of pro-inflammatory cytokines/chemokines, such as IFN-γ, TNF-α, CXCL9, and CXCL10, and increased interferon regulatory factor 1 (IRF1) and programmed death-ligand 1 (PD-L1) expression, resulting in an enhanced anticancer immune response against primary and lung metastatic B16F10 melanoma compared to untreated controls [113]. Tumor destruction was caused by macrophages, DCs, T cells, and NK cells that recognized antigens naturally seen in melanoma, and elevated IFN-γ in the tumor site. In the same study, they also examined the anti-cancer effect of BG34-200 on a transplanted metastatic osteosarcoma cell line in a murine model. It also resulted in immune-boosting activity and diminished tumor burden 56 days after treatment.

Recently, a soluble form of β-glucan (Imprime PGG), which acts as a PAMP, has been developed. Monocyte treatment with Imprime resulted in enhanced production of M2 macrophages and dendritic cells, along with higher expression levels of PD-L1 and CD86, both of which can potentiate the activity of anti PD-1 antibodies. To test this hypothesis, Qiu et al. used Imprime and anti-PD-1 antibodies in combination in a synergistic tumor model. As anticipated, the median tumor volume in the combination group was lower than in animals treated with anti-PD-1 antibodies alone (172 mm^3^ vs. 936 mm^3^, respectively) [115,139]. It is important to note that for Imprime to act efficiently, the prior presence of anti-β-glucan antibodies is required; thus, measuring the pre-treatment levels of these circulating antibodies may help to determine the patients most likely to benefit from this treatment [114].

Taken together, these observations provide evidence that β-glucan is efficacious as an anti-cancer treatment, pointing to its potential therapeutic role when used in combination. Furthermore, tumor microenvironment modulation represents a novel area of treatment that should be investigated.

#### 4.1.2. Probiotics as Adjuvant Therapy

Probiotics were shown to induce tumor cell apoptosis in vitro, and inhibition of tumor cell proliferation and metastasis. Moreover, probiotics improved tumor conditions by modifying the tumor microenvironment in animal models, and consequently boosted the anti-cancer immune response and developed favorable conditions for successful treatment with anti-cancer agents. However, most of the ongoing research on probiotics and cancer mainly focuses on gastrointestinal tumors, giving limited attention to other tumor types. Thus, the underlying mechanisms by which they can inhibit the growth of other tumor types require more exploration.

Chen et al. showed that *Saccharomyces boulardii* CNCM I-745, a probiotic microorganism belonging to the *Saccharomyces cerevisiae* species [140], inhibited the epidermal growth factor receptor (EGFR)–MAPK/ERK kinase (MEK)–ERK signaling network and pro-apoptotic actions in tumor cells by suppressing Akt, a central actor of the cell cycle, thereby regulating the inflammatory responses and suppressing the gut cancer expansion [116]. In this study, the authors reported an exciting observation that the probiotic yeast *S. boulardii* blocks vascular endothelial growth factor receptor (VEGFR) signaling and inhibits angiogenesis both in vitro and in vivo.

The enhanced anti-cancer effect observed with a combined probiotic–chemotherapy treatment modality is not only limited to the administration of beneficial fungi. Chen et al. investigated this effect using *Lactobacillus paracasei* GMNL-133 and *Lactobacillus reuteri* GMNL-89 in combination with the anticancer agent gemcitabine. Multi-strain probiotic administration in a murine model in conjunction with gemcitabine was associated with a greater reduction in the growth rate of pancreatic intraepithelial neoplasia compared to gemcitabine-only treatment and no treatment [117]. Such effects may be of value to boosting the action of anti-cancer therapies, thereby facilitating the use of lower doses, and consequently helping to reduce the undesirable side-effects of these drugs. However, it is important to note that administration of these microorganisms as an adjuvant treatment should be considered carefully, especially in the case of immunocompromised and/or critically ill subjects, such as cancer patients. These patients have higher risks of systemic infection compared to healthy individuals, and treatment with microorganisms may increase the mortality rate [141,142].

#### 4.1.3. Effect on Radiotherapy

The effect of dysbiosis on the radiation therapy response has been recently reported by Shiao et al., who showed that responsiveness to radiation therapy was enhanced following antibiotic-mediated depletion or gnotobiotic exclusion of fungi. On the other hand, depletion of bacteria reduced responsiveness. Furthermore, a negative association was noted between increased intra-tumoral expression of dectin-1, a primary innate sensor of fungi, and survival in patients with breast cancer, and was also required for the effects of commensal fungi in mouse models of radiation therapy [103].

#### 4.1.4. Fungal Metabolites

Another example of potential fungal effects on anti-cancer activity can be found in the endophytic fungi inhabiting the Ginkgo Biloba trees. Gingko Biloba is also known as the maidenhair tree, a species native to China, which was reported to have several medical applications because of its abundant endophytes and various secondary metabolites. Miao et al. isolated 19 strains of endophytic fungi present in Ginkgo Biloba and determined their anticancer effects. At a test concentration of 200 μg/mL, crude extracts from 45.9% of these fungal cultures led to greater than 50% anticancer activity [120].

Additionally, He et al. successfully isolated nine strains of endophytic fungi from the leaves of Ginkgo biloba. Among them, strains J-1, J-2, and J-3 were found to produce podophyllotoxin. These secondary metabolites demonstrated marked inhibition of HeLa cell proliferation, promoted their apoptosis, and blocked their migration. Furthermore, these metabolites significantly attenuated the growth of HeLa tumors implanted in mice [121].

Several microbial enzymes may inactivate the anti-cancer agent, resulting in an overall reduction in the therapeutic efficacy or tolerability of the drug. For example, *Bacteroides* spp., a normal inhabitant of the gut microbiome, can accelerate the conversion of sorivudine (synthetic analogue of thymidine) into bromovinyluracil (BVU), which accumulates in the blood, resulting in increased toxicity in patients taking oral UFT (a combination of ftorafur, a 5FU prodrug, with uracil) [143].

In another study, the overexpression of the enzyme cytidine deaminase was associated with reducing gemcitabine (one of the most prescribed agents for patients with pancreatic ductal adenocarcinoma) into its inactive metabolite [122]. Thus, modification of the microbial environment by reducing the numbers of these microorganisms would enhance the activity of gemcitabine. In line with this hypothesis, mycobiome ablation enhanced the activity of gemcitabine, as reported by Aykut et al. [26]. Furthermore, a protective effect against tumor growth was observed in slowly progressive and invasive models of pancreatic ductal adenocarcinoma (PDA) used in that study [26]. On the other hand, repopulation with *Malassezia* species was demonstrated to accelerate oncogenesis. However, the mechanisms underpinning these observations are still incompletely understood. Thus, further investigations are required to explore the potential of targeting fungi to enhance responsiveness to traditional cancer therapeutics or immunotherapy.

Another interesting study has investigated the underlying anti-cancer mechanism of schizophyllan (SPG) in murine cancer models [123]. SPG is a neutral extracellular polysaccharide produced by the fungus *Schizophyllum*, noted for its anti-cancer activity, especially against lung, cervical, and gastric cancers [124,125]. The anti-cancer effect of SPG was reported to be dependent on its interaction with dectin-1, a cell surface receptor for 1,3-β-glucan (a component of the cell wall in many fungi). Administration of dectin-1 antibodies resulted in reduced SPG activity [123], highlighting the potential role that fungi may play in the treatment of cancer.

#### 4.1.5. Other Promising Treatments

Stem cell transplantation (SCT) is one of the evolving treatment modalities for a wide range of medical conditions, including cancer treatment (e.g., hematologic malignancy) [144]. A well-documented complication of this treatment is graft-versus-host disease (GVHD), where the donor’s immune cells perceive the recipient tissue as foreign, resulting in multisystem organ damage and increased incidence of infection [145]. Several mechanisms can contribute to the development of GVHD, including gut microbial translocation caused by a transplantation conditioning regimen, and consequently, activation of certain immune cells, such as Th17, resulting in a proinflammatory reaction and organ damage [146,147]. With regard to this outcome, an association between *Candida* colonization of SCT recipients and the incidence of GVHD has been reported [126]. Colonization with *Candida* was found to be accompanied by a significant increase in the incidence of acute GVHD compared to non-colonized patients [126]. Furthermore, anti-microbial chemotherapy targeted toward intestinal anaerobic bacteria in SCT patients showed a significant reduction in the severity of acute GVHD and supports the hypothesis that the intestinal anaerobic bacterial microflora contribute to the pathogenesis of acute GVHD following bone marrow transplantation [119].

In light of these observations, therapeutic approaches modifying gut microbiota could be used to decrease the likelihood of complications in patients receiving SCT or bone marrow transplantation while preserving some benefits of this anticancer treatment modality, such as the graft versus leukemia effect [148]. An example of a gut-modifying treatment is fecal microbiota transplantation (FMT), which has been studied as a potential approach for the prevention of CRC [149,150]. Furthermore, a recent clinical trial (NCT03341143) showed that FMT improved the response to anti-PD-1 therapy in melanoma patients [118]. The combined administration of FMT and anti-PD-1 was shown to modify the tumor microenvironment by increasing microbial communities that were reported to enhance the efficacy of anti-PD-1. The investigators attributed the failure of this combination to the absence of these microorganisms in the transplanted feces. In support of this hypothesis, a study speculated that the success rate of patients with *Clostridium difficile* infection treated with FMT is related to the microbial composition of the donor stool. Essentially, higher relative abundances of *Saccharomyces* and *Aspergillus* were reported to correlate with effective FMT, whereas reduced FMT efficacy was related to *C. albicans* overgrowth [151]. Thus, an important aspect of the FMT treatment modality is the composition and the source of the feces being transferred, as these may significantly impact the response following the FMT.

Several strategies to modulate FMT have been employed, including probiotics, prebiotics, postbiotics, and antibiotics. Although these strategies provide promising results mechanistically by adjusting the microbiota, modulating the innate immune system, enhancing gut barrier function, preventing pathogen colonization, and exerting selective cytotoxicity against tumor cells, it is crucial to highlight that they are not without risks and controversies, as they may introduce clinical complications [152]. Further evaluation of this approach is needed.

### 4.2. Effect of Anticancer Therapy on the Mycobiome

The relation between chemotherapy and anti-cancer targeted therapy is bidirectional. In other words, in addition to the microbiota affecting therapeutics, these drugs can also cause severe disruption to the microbiome’s composition, leading to dysbiosis [153]. For example, studies have shown that anticancer drugs can lead to the reduction of beneficial microorganisms, thereby negatively impacting gut-protective mechanisms. This, in turn, may accelerate the development of chemoresistance [153].

Another important category of drugs that are usually prescribed to cancer patients is antibiotics. Our group recently showed that broad-spectrum antibiotics (e.g., minocycline), unlike narrow-spectrum antibiotics, lead to microbial dysbiosis [154]. Such an effect may alter treatment outcomes and contribute to drug resistance [155]. For instance, patients with lung or renal cancer who were given antibiotics within one month of immune checkpoint inhibitor therapy had worse clinical outcomes [156].

Similarly, in a study by Robinson et al., the type and intensity of chemotherapeutic agents were found to cause specific alterations in the oral mycobiome. Following chemotherapeutic administration, analysis of the mycobiomes from oral samples demonstrated reduced α-diversity in subjects receiving high-intensity chemotherapy, compared to those receiving low-intensity regimens. Additionally, patients on high-intensity remission induction chemotherapy had a significant decrease in *Malassezia* levels compared to those on lower intensity regimens [105]. Interestingly, the relative abundance of *Candida* was significantly higher in patients that contracted infections prior to neutrophil recovery, whereas significantly higher relative abundance of *Fusarium* was found among patients who did not get an infection [105]. A similar study by Hong et al., investigated the association between disruption of the oral microbiome and chemotherapy-induced mucositis; however, this study focused mainly on the alteration of the bacteriome only [106].

Additionally, Ghannoum et al. studied the effects of chemotherapy, X-irradiation, and a combination of both on yeast adhesion to buccal epithelial cells (BEC) in vitro. The growth of three *Candida* spp., in the presence of 8/11 antineoplastic drugs, reduced the adherence of the isolates tested (30–61% reduction compared to untreated control values). The adhesion of *C. albicans* to BEC was reduced by 31–53% when exposed to various radiation levels [96]. The mechanism(s) by which antineoplastic agents inhibit *C. albicans* adhesion to BEC are unknown. Two mechanisms may be responsible. The first one involves *Candida* species’ absorption of antineoplastic agents. These modifications may be mirrored in the diverse modifications (depending on the agent) of the outer surface structures known to mediate adherence (such as alterations in adhesins). Additionally, at the concentrations utilized, these agents may limit the synthesis and expression of yeast adhesins. The second mechanism could be that these substances are not delivered by yeasts but are instead adsorbed at the cell surface, masking adhesins and thereby preventing adherence.

## 5. Future Perspectives

The growing evidence obtained from several clinical and preclinical studies highlights the importance of considering combining conventional chemotherapy or radiotherapy with a microbiome-modulating regimen, as these are potentially effective strategies for managing cancer. A clear fungal signature showing the involvement of the fungal community in various types of tumors calls for broadening the scope of microbiome studies beyond bacteria. Additionally, interkingdom interaction between microbial communities has been reported in several studies, and all agree that multispecies communities contribute collectively rather than individually to the development of dysbiosis or maintenance of a healthy community [7]. Thus, although most studies focus only on the bacterial microbiome, changes in the bacteriome profile will inevitably be accompanied by changes in the mycobiome profile. Based on this, microbiome work should focus on understanding the complex and dynamic relation between these communities (e.g., bacteria, fungi, and viruses) which will potentially facilitate optimizing these interactions for beneficial applications.

In conclusion, despite the progress to date, the microbiome is an area of research still in its nascent stages, and the relation between cancer and microbiome, with an emphasis on newly emerging mycobiome connections, should be actively studied. Moreover, comprehensive data are needed to fully understand the potential direct anti-cancer effects of the mycobiome and how to utilize it in future targeted therapies. Studies similar to those demonstrating how the microbiome alters the responses of checkpoint inhibitors should begin to incorporate the mycobiome and its potential roles in host immunomodulation and response to improve the treatment outcomes and quality of life of cancer patients.

## Figures and Tables

**Table 1:** Published studies investigating the relationship between the fungal community and cancer.

Type of Cancer	Evidence of Mycobiome Implication	Author/Year
Colorectal cancer	Increased *Ascomycota*, and *Basidiomycota*.	Luan et al., 2015 [21] Gao et al., 2017 [22] Richard et al., 2018 [23]
	Decreased fungal diversity in polyps compared to adjacent tissue.	Luan et al., 2015 [21] Gao et al., 2017 [22] Coker et al., 2019 [8]
	Increased ratio of *Ascomycota* to *Basidiomycota* in CRC patients.	Coker et al., 2019 [8] Gao et al., 2017 [22]
	Increased opportunistic fungi; *Trichosporon* and *Malassezia*, which could be implicated in the progression to CRC.	Gao et al., 2017 [22]
	Distinctive sets of proteins secreted by *Schizosaccharomyces pombe* in patients’ stool samples. Increased *Saccharomycetales* in advanced adenoma versus non-advanced samples.	Chin et al., 2018 [24]
	CARD9 deficient mice exhibit fungal dysbiosis resulted in increased CRC tumor loads.	Luan et al., 2015 [21]
	Treatment with fluconazole suppressed tumor growth in mice.	Wang et al., 2018 [25]
Pancreatic ductal adenocarcinoma (PDA)	In comparison to the normal pancreas, PDA tumors have a 3000-fold increase in fungi. In mice and humans, the fungal community infiltrating PDA was enriched in *Malassezia*. In both slowly progressive and invasive PDA models, fungal elimination with amphotericin B was tumor-protective, while re-population with *Malassezia*–but not *Candida*, Saccharomyces, or Aspergillus–promoted oncogenesis. Connection of MBL, that attaches fungal wall glycans to activate the complement pathway, was needed in the promotion of malignancy. Tumor growth was inhibited by MBL or C3 deletion in the extra-tumoral region or C3aR knockdown in tumor cells. Pathogenic fungi may promote PDA by inducing MBL, which activates the complement system.	Aykut et al., 2019 [26]
Gastric Cancer	Unique fungal profile was observed in gastric cancer biopsies. *C. albicans*, *Arcopilus aureus*, *and Fusicolla* spp. were enriched in GC compared to the control, whereas *C. glabrata*, *Aspergillus montevidensis*, *Saitozyma* and *Penicillium* were depleted.	Zhong et al., 2021 [27]
Head and neck SCC/Oral SCC	*C. albicans*, *C. etchellsii*, *Hannaella*, and *Gibberella* were prevalent in OSCC specimens, while *Altenaria* and *Trametes* were observed in larger abundance in polyps’ specimens	Perera et al., 2017 [28] Vesty et al., 2018 [29] Shay et al., 2020 [30] Makinen et al., 2018 [31]
	Polyp specimens dominated by *Malassezia restricta* and *Aspergillus tamarii*.	Vesty et al., 2018 [29]
	Marked expression of IL-1, IL-6, and IL-8 by oral cancer cells which are usually associated with *C. albicans.*	Dongari-Bagtzoglou, and Kashleva, 2003 [32] Vesty et al., 2018 [29] Arzmi et al., 2019 [33]
	Compared with healthy controls, *Schizophyllum commune* was significantly lower in HNSCC patients.	Shay et al., 2020 [30]

**Table 2:** Summary of the evidence that supports the potential use of fungal signature as a tumor biomarker.

Type of Cancer	Finding	Reference
**I. Cancers of the Gastrointestinal tract**		
Colorectal cancer	Increase in the *Ascomycota*: *Basidiomycota* ratio and enrichment of *Microascaceae* and *Sordariaceae*_spp. in CRC patients compared to healthy subjects. Increase in *Microbotryomycetes*, *Sordariomycetes*, *Microascaceae*, *Sordariales*, *Lasiosphaeriaceae*, and *Microascales*, with a decrease in the abundance of *Pleosporaceae* and *Alternaria* was detected in late-stage CRC patients.	Gao et al., 2017 [22]
	*Basidiomycota* was significantly enriched in adjacent biopsy samples. The phylum Chytridiomycota was significantly enriched in adenomas compared with adjacent biopsy samples. The order *Saccharomycetales* and phylum *Basidiomycota* organisms were significantly enriched in advanced versus non-advanced adenoma tissue biopsies.	Luan et al., 2015 [21]
	The abundance of *Candida*, *Saccharomyces*, and *Ascomycete* was elevated in patients with colonic polyps.	Chen et al., 2019 [94]
	Enrichment of *Rhodotorula*, *Malassezia*, and *Acremonium* with reduction of *Saccharomycetes*, especially *S. cerevisiae*, in fecal samples from CRC patients.	Coker et al., 2019 [8]
	Proteins secreted by *Schizosaccharomyces pombe* in the stool samples of CRC patients.	Chin et al., 2018 [24]
Pancreatic ductal adenocarcinoma (PDA)	PDA patients were found to have a 3000-fold increase in fungal abundance compared to a cohort of subjects with a healthy pancreas. Anti-fungal medication in a mouse model of PDA protected against tumor progression, whereas repopulation with *Malassezia* resulted in rapid growth of pancreatic tumors.	Aykut et al., 2019 [26]
Head and neck SCC/Oral SCC	*C. albicans* was the most commonly isolated species among OSCC in most patients.	Mäkinen et al., 2018 [31]
	*Malassezia* was reduced in abundance in patients with HNSCC.	Shay et al., 2020 [30]
	*Rhodotorula*, *Geotrichum*, and *Pneumocystis* were only detected in samples obtained from the cancer patient samples. *Fonsecaea* was found in both cancer samples and cancer-adjacent tissue from the same patients but not in oral tissue from healthy subjects.	Banerjee, Sagarika et al., 2017a [95]
	Glomeromycota was the only fungal phylum that was significantly decreased in the tumor group compared to their matched non-tumor tissues. High T-stage tumor samples exhibited an increased richness of both bacteria and fungi at the phylum level compared to low T-stage samples (*p* ≤ 0.047).	Mukherjee et al., 2017 [96]
	Overgrowth of *C. albicans* in oral squamous cell malignant tissue.	Perera et al., 2017 [28]
	Increased concentration of *C. albicans* in the saliva of HNSCC patients.	Vesty et al., 2018 [29]
**II. Cancers beyond the Gastrointestinal Tract**		
Ovarian cancer	Eighteen fungal signatures were detected only in ovarian cancer samples including: *Cladosporium*, with the highest hybridization signal, *Acremonium*, and *Candida*.	Banerjee, S. et al., 2017b [97]
Breast cancer	The highest fungal diversity was detected in ER+ samples, while HR+ samples had the least fungal diversity. *Arthroderma* accounted for the highest average hybridization signal. *Candida*, *Cryptococcus*, *Mucor*, *Penicillium*, *Trichophyton*, and *Trichosporon* were observed in all cancer samples.	Banerjee, S. et al., 2021 [98]
Melanoma	α-diversity of the fungal community revealed significantly higher richness in melanoma patients compared to controls. Reduction in the fungal diversity as the disease progressed. *Saccharomycetales* was enriched in melanoma patients. One hundred and eighty zOTUs (including 63 fungal zOTUs) were enriched in melanoma patients compared to controls. Out of these zOTUs, 162 were mainly enriched in patients with in situ melanoma. Analysis of the gut microbiota revealed a significant difference between metastatic and nonmetastatic melanoma patients.	Vitali et al., 2022 [99]

**Table 3:** Summary of the evidence demonstrating mycobiome–anticancer therapy interaction.

Type of Study	Investigated Agent/Modality	Finding	Reference
In vitro and In vivo—Murine tumor model	β-glucan	Oral administration can boost phagocytes’ tumoricidal activity against iC3b-opsonized cancer cells.	(Hong et al., 2004) [107]
In vitro and In vivo—Murine tumor model		Oral administration decreased the tumor burden by inducing the transition of immunosuppressive M2 macrophages into inflammatory M1 macrophages via dectin-1 receptor.	(Liu et al., 2015; Wang et al., 2015) [68,108]
Clinical Trial		Oral administration of two 10-mg capsules of soluble β-glucan derived from *S. cerevisiae* enhanced the proliferation and activation of peripheral blood monocytes with no clinical adverse effects (Mean monocyte count increased from 326 ± 124/mm^3^ to 496 ± 194/mm^3^ on day 15 post-administration *p* = 0.015).	(Demir et al., 2007) [109]
In vitro and In vivo—Murine tumor model		Intravenous administration with β-glucan derived from mutated *S. cerevisiae* strain suppressed cancer cell proliferation in a dose-dependent manner. Pretreatment of mice with the same type of β-glucan, 2 days before inoculation with the tumor cells, enhanced mice survival time. Enhanced pro-inflammatory cytokine production and anticancer activity of peritoneal macrophages, and increased natural killer (NK) cell cytotoxicity.	(Yoon et al., 2008). [110]
In vivo—Murine tumor model		Prophylactic intravenous administration of β-glucan, derived from mutated *S. cerevisiae*, in combination with cisplatin, had better efficacy compared to the chemotherapy agent alone.	Kim et al., 2010 [111]
In vitro and In vivo—Murine tumor model		Oral administration of β-glucan extracted from *S. cerevisiae* suppressed the development of melanoma in a dose-dependent manner.	Vetvicka and Vetvickova, 2015 [112]
In vitro and In vivo—Murine tumor model		Systemic administration (intraperitoneal or intranasal) of oat-derived β-(1-3)—(1-4)-glucan resulted in activation of M1-type macrophage, production of pro-inflammatory cytokines (such as IFN-γ, TNF-α, CXCL9, and CXCL10), IRF1, and PD-L1 expression, resulting in enhanced anticancer immune response compared to untreated controls.	Zhang et al., 2018 [113]
In vitro		Monocyte treatment with Imprime (soluble type of β-glucan) resulted in enhanced production of M2 macrophages and dendritic cells with higher expression of PD-L1 and CD86, both of which can potentiate the activity of anti PD-1 antibodies. For Imprime to act efficiently, prior presence of anti-β-glucan antibodies is required.	Chan et al., 2016 [114]
In vivo—Murine tumor model		In a synergistic tumor model, Imprime and anti-PD-1 antibodies combination group had a lower median tumor volume compared to the anti-PD-1 antibodies alone- treated group (172 mm^3^ vs. 936 mm^3^, respectively).	Qiu et al., 2016 [115]
In vivo—Murine tumor model	Probiotics	*Saccharomyces boulardii* inhibited the EGFR-MEK-ERK signaling network and pro-apoptotic actions in tumor cells by suppressing Akt, thereby regulating the inflammatory responses and suppressing the gut cancer expansion. *S. boulardii* blocks vascular endothelial growth factor receptor (VEGFR) signaling and inhibits angiogenesis both in vitro and in vivo	Chen, X. et al., 2009 [116]
In vivo—Murine tumor model		Administration of *Lactobacillus paracasei* and *Lactobacillus reuteri* in combination with the anticancer agent gemcitabine in murine model was associated with a more reduction in the growth rate of pancreatic intraepithelial neoplasia compared to gemcitabine only treated group and untreated controls.	Chen, S.M. et al., 2020 [117].
Human subject and In vivo—Murine tumor model	Bacteriome and mycobiome modulation.	Responsiveness to radiation therapy was enhanced following antibiotic-mediated depletion or gnotobiotic exclusion of fungi. Depletion of bacteria reduced responsiveness. A negative association was noted between increased intra-tumoral expression of Dectin-1 and survival in patients with breast cancer, and was also required for the effects of commensal fungi in mouse models of radiation therapy	Shiao et al., 2021 [103]
Clinical Trial		FMT improved the resistance to anti-PD-1 therapy in melanoma patients.	Davar et al., 2021 [118]
Human subject and In vivo—Murine tumor model	Mycobiome modulation.	Mycobiome ablation: Enhanced the activity of gemcitabine. Offered a protective effect against tumor growth in slowly progressive and invasive models of pancreatic ductal adenocarcinoma. Repopulation with *Malassezia* species accelerated oncogenesis.	Aykut et al., 2019 [26]
Clinical trial	Bacteriome Modulation	Antimicrobial chemotherapy targeted toward intestinal anaerobic bacteria in SCT patients showed a significant reduction in the severity of acute GVHD following bone marrow transplantation.	Beelen et al., 1999 [119]
In vitro	Fungal metabolites	Crude extracts of several endophytic fungal strains present in Ginkgo biloba exhibited anticancer activity at a test concentration of 200 μg/mL.	L. Miao, 2009 [120]
In vitro and In vivo—Murine tumor model		Three strains of endophytic fungi from the leaves of Ginkgo biloba were found to produce podophyllotoxin. Their metabolites demonstrated: marked inhibition of HeLa cell proliferation, promoted their apoptosis, blocked their migration, and significantly attenuated the growth of HeLa implanted tumors in mice.	He et al., 2020 [121]
Human subject and In vivo—Murine tumor model		Overexpression of the enzyme cytidine deaminase is associated with reducing gemcitabine into its inactive metabolite.	Geller et al., 2017 [122]
In vitro and In vivo—Murine tumor model		Some *Schizophyllum* species metabolites possess anticancer activity. Administration of dectin-1 antibodies resulted in reduced SPG activity. Schizophyllan anti-cancer effect is dependent on its interaction with Dectin-1.	Ikeda et al., 2007; Zhong et al., 2015; Lopez-Legarda et al., 2021 [123,124,125]
Human subjects	Others	Colonization with *Candida* is accompanied by a significant increase in the incidence of acute GVHD compared to non-colonized patients.	Van Der Velden et al., 2010 [126]

## Data Availability

Not applicable.
